# Advances in Female Germ Cell Induction from Pluripotent Stem Cells

**DOI:** 10.1155/2021/8849230

**Published:** 2021-01-13

**Authors:** Maisumu Gulimiheranmu, Xinjie Wang, Junmei Zhou

**Affiliations:** Department of Central Laboratory, Shanghai Children's Hospital, Shanghai Jiao Tong University, 1400 Beijing Road West, Shanghai 200040, China

## Abstract

Germ cells are capable of maintaining species continuity through passing genetic and epigenetic information across generations. Female germ cells mainly develop during the embryonic stage and pass through subsequent developmental stages including primordial germ cells, oogonia, and oocyte. However, due to the limitation of using early human embryos as *in vivo* research model, *in vitro* research models are needed to reveal the early developmental process and related mechanisms of female germ cells. After birth, the number of follicles gradually decreases with age. Various conditions which damage ovarian functions would cause premature ovarian failure. Alternative treatments to solve these problems need to be investigated. Germ cell differentiation from pluripotent stem cells *in vitro* can simulate early embryonic development of female germ cells and clarify unresolved issues during the development process. In addition, pluripotent stem cells could potentially provide promising applications for female fertility preservation after proper *in vitro* differentiation. Mouse female germ cells have been successfully reconstructed *in vitro* and delivered to live offspring. However, the derivation of functional human female germ cells has not been fully achieved due to technical limitations and ethical issues. To provide an updated and comprehensive information, this review centers on the major studies on the differentiation of mouse and human female germ cells from pluripotent stem cells and provides references to further studies of developmental mechanisms and potential therapeutic applications of female germ cells.

## 1. Introduction

Currently, female infertility caused by various reasons is becoming an exacerbating reproductive problem. Assisted reproductive technology (ART) is an effective treatment for non-germ cells (GCs-) caused infertility. However, infertility caused by GCs abnormalities has not yet had a good alternative treatment [1]. Treating infertility among these patients requires a precisely detailed understanding of female GCs differentiation and pathological defects which occurred in abnormal female GCs. However, female GCs formation mainly occurs during the embryonic stage. Due to the limited acquisition and ethical inhibition to early human embryos for research purpose, early female GCs development have not been revealed deliberately [2]. Therefore, establishing an appropriate *in vitro* model is necessary for the investigations on female GCs development and fertility reconstruction.

A mouse model is most commonly used to study mammalian female GCs formation, specialization, and differentiation [3]. Significant achievements have been gained in inducing mouse female GCs from pluripotent stem cells (PSCs) which provide remarkable references for reconstructing human female GCs *in vitro* from PSCs [4–8]. Both embryonic stem cells (ESCs) and induced pluripotent stem cells (iPSCs) have competence for self-renewal and multilineage differentiation including female GCs [9–12]. However, the induction protocols are slightly different between mouse PSCs and human PSCs based on the differences of *in vivo* female GCs formation between mice and humans [4–8].

## 2. Female Germ Cells Development *In Vivo*

**Figure 1:**
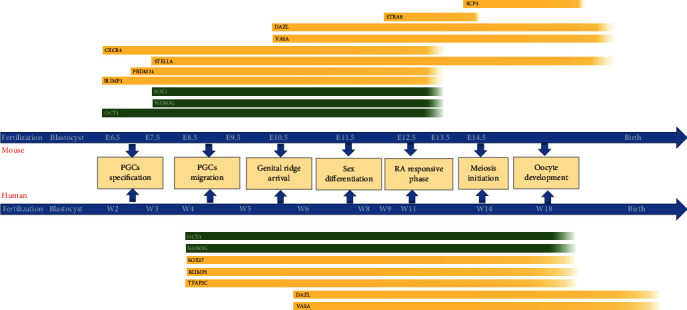
Schematic of female germ cell development and key gene expressions during the development. Developmental timelines and stages of mouse and human female germ cell development are shown in the center. PGCs undergo specification and migration then arrive at the genital ridge. After sex differentiation, PGCs subsequently undertake RA responsive phase, meiosis initiation, and oocyte development. Key gene expressions corresponding to different developmental stages are shown in yellow bars.

Mouse primordial germ cells (PGCs) were first discovered at the posterior end of the primitive streak in the extraembryonic mesoderm at embryonic day 6.25 (E6.25) [13], followed by PGC specification at E7.25 and migration at E9.5. At around E10.5, PGCs reach the genital ridge and enter embryonic gonads at E11.5 [14]. The ultimate sexual fate is not only regulated by the chromosomal constitution but also by the gonadal somatic cells [14]. Before interweaving with the signals from embryonic gonadal somatic cells, PGCs are “bipotential,” which means PGCs could adapt either male or female fate [14]. After colonizing embryonic ovaries, PGCs begin sex differentiation at E12.5 and develop into oogonia at E13.5 [15]. Afterward, at E14.5, oogonia enter meiosis I and form primary oocytes which are arrested at the meiosis I diplotene stage until ovulation. At around birth, the primary oocytes were surrounded by granulosa cells and sequentially generate primordial, primary, secondary, and antral follicles [16]. Primary oocytes complete meiosis I around six weeks after birth and form secondary oocytes. Secondary oocytes are ovulated and arrested at metaphase of meiosis II (MII) before fertilization. MII oocytes are considered as the functional oocytes that could be fertilized with spermatozoa (Figure 1).

**Figure 2:**
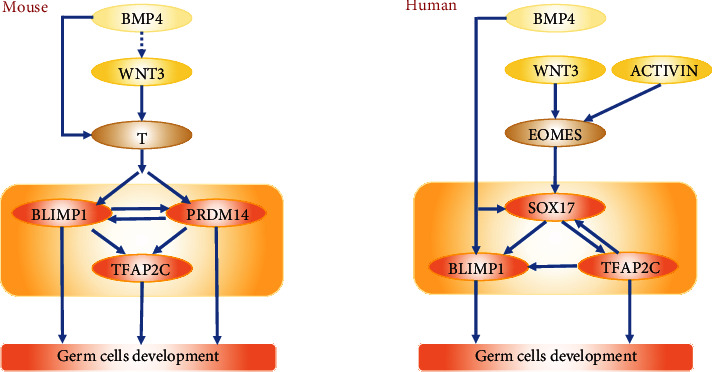
Transcriptional regulatory network models for mouse and human PGCs specification. Full and dashed arrows indicate direct and indirect regulations, respectively.

Mouse PGCs differentiation occurs under the regulation of sequential transcription factors (TFs) (Figure 2). Bone morphogenetic protein (BMP) and Wingless/Integrated (WNT) pathways trigger a set of downstream TFs [17]. BMP4 activates WNT3, which is located at the upstream of a mesodermal TF- BRACHYURY (T) [17]. T activates critical early GCs markers BLIMP1, PRDM14, and TFAP2C synergistically with BMP4 [17]. BLIMP1 is expressed in the precursors of mouse PGCs, induces PRDM14 and TFAP2C, activates the germline pathway, and robustly represses a somatic mesodermal pathway [15, 18]. PRDM14 is specifically expressed in mouse PGCs. Studies showed PRDM14 is essential for epigenetic reprogramming in mouse PGCs [19]. Thus, these interactions between TFs are essential for the subsequent differentiation of female GCs. During specification, at around E7.25, mouse PGCs express pluripotency markers OCT4, NANOG, SOX2, KLF2, and PGCs-specific markers SSEA1 and STELLA (Figure 1) [18, 20]. OCT4, which is critical for the specialization and maintenance of mouse PGCs exhibited high expression until sex differentiation [15]. SOX2 directly contributes to the survival and proliferation of mouse PGCs [21]. Migratory mouse PGCs mainly express SSEA1 and CXCR4 [19, 20]. DAZL and VASA begin to be expressed when sex differentiation is imminent. DAZL is considered as a germ cell-intrinsic competence factor, which is necessary for receiving signals from extrinsic factors in embryonic gonads. After sex differentiation, meiosis I is initiated by retinoic acid (RA) at around E12.5. RA induces premeiotic gene STRA8 and meiosis-associated gene REC8 expression in embryonic ovaries. STRA8 upregulates synaptonemal complex protein3 (SCP3) and DMC1, both of which represent meiosis initiation at E13.5 [22]. In conclusion, these stage-specific markers not only provided insights into GCs developmental mechanisms but also offered specific markers for assessing differentiated cells during female GCs development, as well as inducing female GCs differentiation through overexpression.

In humans, PGCs differentiation is similar in broad strokes with mouse PGCs, but varies in developmental timing (Figure 1), TFs interactions, and certain specific markers (Figure 2). Human PGCs were first identified by Fuss and Felix in the dorsal wall of the yolk sac endoderm at developmental week 3 (Wk3) [23, 24]. In later studies, researchers detected human PGCs are specified in the posterior epiblast of early postimplantation embryos approximately at Wk2 [25, 26]. Then, human PGCs start migration around Wk4 [27, 28] and enter genital gonads around Wk5-6 [29]. During Wk6-8, PGCs sex-differentiated with the gonadal somatic cells in embryonic ovaries [30]. With the interactions between PGCs and gonadal somatic cells, oogonia cells are formed at Wk9 and respond to RA signals around Wk11 to differentiate into primary oocytes at Wk14 [26, 31]. Afterward, primary oocytes assembled into primordial follicles with a layer of granulosa cells [32]. At birth, there are approximately 300,000 primordial follicles, and this number mostly declines with age after birth [33]. The subsequent folliculogenesis, completion of meiosis I, and generation of MII oocytes proceeded in a mostly analogous way with different point-in-time to mice.

Human PGCs specification occurred under BMP4, EOMES, SOX17, BLIMP1, and TFAP2C transcriptional network approximately similar to that of mice (Figure 2). EOMES, which is a critical factor in human mesodermal precursor cells, is located at the downstream of Activin and WNT signaling, meanwhile at the upstream of SOX17, BLIMP1, and TFAP2C [34]. SOX17, an essential specifier of human PGCs, also activates BLIMP1 and TFAP2C, both of which in turn activate the germline pathway and repress mesoderm, endoderm, and neural pathway [34]. The crucial mesodermal TF-T during mouse PGCs formation, however, is not essential for humans [34]. KLF4, a naive pluripotency factor, is expressed in human PGCs while in mice repressed by BLIMP1 [35]. In contrast, SOX2 and PRDM14, which are critical for mouse PGCs differentiation, are not highly expressed in human PGCs [7, 36]. Migrating human PGCs mainly express early GCs markers BLIMP1, TFAP2C, and SSEA1 as well as pluripotency markers OCT4 and NANOG. At the end of migration, DAZL and VASA are expressed at a lower level [26]. RA responsive genes STRA8, RDH10, and CYP26A1 begin to express as early as Wk11, indicating the imminent initiation of meiosis. The meiotic prophase female GCs mainly express SCP1, TEX12, and SPO11. The primary oocytes are characterized by ZP1-3, NOBOX, and OOSP2 expressions [26].

These abovementioned transcriptional factors and female GCs markers corresponding to different developmental stages provided important references for the establishment of the differentiation system *in vitro*. Meanwhile, the established *in vitro* models, in turn, elucidated the abovementioned mechanisms during female GCs formation. Continuing the described studies will elucidate precisely how mouse and human PSCs are induced into female GCs, respectively.

## 3. Female GCs Induction from PSCs *In Vitro*

The most commonly used PSCs are ESCs and iPSCs. Mouse and human ESCs were derived from inner cell mass (ICM) of the blastocyst in 1981 and 1998, respectively [37, 38]. ESCs have competence for self-renewal and multilineage differentiation potential to cells of three germ layers. However, the establishment of human ESCs needs to destroy early human embryos, thus resulting in ethical concerns. Also, xenotransplantation of ESCs-derived cells may probably cause immunological rejection. These concerns were relieved by the establishment of iPSCs. In 2006, scientists induced mouse iPSCs through the introduction of four key transcription factors—OCT3/4, SOX2, KLF4, and c-MYC—into mouse adult fibroblasts [9]. Subsequently, human iPSCs were generated from adult human fibroblasts [10, 39]. These iPSCs have become attractive alternatives of ESCs for their analogous biological characteristics to ESCs in cell morphology, gene expressions, and surface antigens. iPSCs were acquired *in vitro* without damaging early embryos, which could dispel ethical concerns about ESC acquisition and application. Furthermore, autologous cell transplantation derived from individual iPSCs avoids allogeneic immune rejection from ESCs. More importantly, they are also capable of differentiating into multilineage cells including female GCs [10]. Therefore, PSCs were studied to generate female GCs, especially iPSCs were regarded as relatively ideal stem cell sources for regenerative medicine.

Generally, ESCs/iPSCs were induced into the germline pathway through spontaneous differentiation, direct induction with some cytokines, or overexpression of germline-specific genes. Induced female GCs were identified by the expression of stage-specific markers as well as the morphology or the functions. Scientists achieved great advances in inducing female GCs from PSCs [4, 6–8]. The induction schemes are slightly different between mouse and human PSCs based on their female GCs development discrepancies.

### 3.1. Female GCs Induction from Mouse PSCs *In Vitro*

Studies about mouse female GCs induction from PSCs acquired significant achievements in the recent two decades (Table 1). *In vitro* female GC induction was first evidenced from mouse ESCs in 2003 [40]. In this study, mouse ESCs were spontaneously differentiated in suspension condition without LIF and feeder cells. On the 12th day of culture, high GFP+/VASA+ expressions were detected in large colonies, which most likely represent postmigratory PGCs. These GFP+/VASA+ PGCs spontaneously formed oogonia-like cells, entered meiosis around the 16th day, and produced oocyte-like cells up to 20% at around the 26th day. Oocyte-like cells were characterized by zona pellucida (ZP) like coats, oocyte markers ZP2 and ZP3 expression. Subsequently, they formed small follicle-like cells (FLCs), which could be cultured into organized structures morphologically similar to primordial follicles. At around the 43rd day, some oocytes that completed meiosis I even could form blastocyst-like structures through parthenogenic activation [40]. These results indicated that mouse ESCs have the potential to spontaneously proceed beyond sex determination and differentiate into mouse female GCs approximately following the development phase and timing *in viv*o [41]. This pioneering study has revealed that mouse ESCs could be a new cell source for oocyte generation. However, in this study, oocyte-like cells have not been evidenced as mature oocytes. Besides, they were generated without directed differentiation and the induction efficiency is rather low. The addition of several growth factor signals was considered to directly differentiate the PSCs to germline and enhance the differentiation efficiency [42]. Researchers collected conditioned medium from testicular cell cultures since testis contain numerous growth factors like BMP4, SCF, LIF, *β*FGF, and GDF9. Mouse ESCs generated embryonic bodies (EBs) in suspension culture and were further induced into oocyte-like cells surrounded by one or two layers of flatted cells which resemble granulosa cells *in vivo*. This indicated testicular cell cultures could provide essential growth factors also for follicle formation [42]. However, in their study, the oocyte-like cells expressed oocyte markers SCP3, ZP3, and FIG*α* but not ZP1 and ZP2, indicating these oocyte-like cells are at an early stage of oocyte growth. Besides, they did not found synapsis despite the SCP3 existence. Regarding oocytes are generated under the interactions between PGCs and gonadal somatic cells *in vivo*, the spontaneous differentiation of oocytes from mouse ESCs was seen as a rare event, and gonadal somatic cells were considered necessary for oocyte-like cell induction [43]. Regarding this, researchers used a two-step method to induce oocyte-like cells from mouse ESCs [43]. First, PGCs were induced through EB formation in 4 days. They cultured mouse ESCs in LIF-free DMEM containing 10% FBS to form EB. EBs expressed OCT4, C-KIT, FRAGILIS, STELLA, and MVH. They sorted SSEA-1 and C-KIT positive cells which represent early PGCs then cocultured with gonadal somatic cells for further 10 days. The differentiated cells expressed female oocyte-specific markers FIG*α*, NOBOX, GDF9, and ZP1-3. However, these markers could not be detected when EBs were cultured alone. This is demonstrating that granulosa cells could enhance the female GCs induction. However, like previous studies [42], oocytes are still arrested at an early meiosis stage even after being cocultured with granulosa cells. Therefore, oocyte growth might require some additional factors that have not been included in these studies [42, 43]. Researchers assumed RA addition might contribute to meiosis completion since it could stimulate STRA8 and REC8 to enter meiosis *in vivo* [44]. Then, mouse ESCs-derived EBs were cultured under RA supplement for 10-15 days [45]. After RA treatment, researchers detected FLCs and presumptive germinal vesicle (GV) oocytes. Furthermore, these GV oocytes could be fertilized with sperms and develop into blastocysts. Thus, RA was confirmed critical for female GCs reconstitution.

In 2009, after the successful establishment of iPSCs, the chimaeric mouse was formed from mouse iPSCs by tetraploid complementation, demonstrating that mouse iPSCs have female GCs competency [46]. Similar to mouse ESCs induction in previous studies, mouse iPSCs were induced into round-shaped oogonia-like cells through EB formation in suspension culture supplemented with RA, BMP4, SCF, EGF, and GDNF [42, 45, 47]. This demonstrated that iPSCs and ESCs could be induced into female GCs through analogous induction methods.

**Figure 3:**
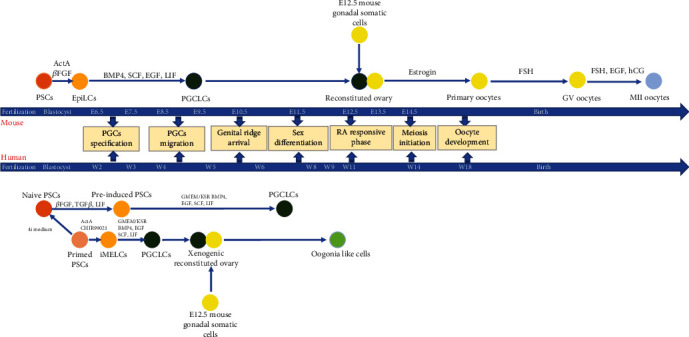
Schematic of the reconstitution of mouse and human female germ cells *in vitro*. Mouse and human female germ cell inductions are described in the upper and lower panel. *In vitro*-induced cells are represented with coloured circles.

The abovementioned studies established some useful approaches for female GCs induction; however, they failed to provide sequential systematic induction protocols with the clear transition from PSCs to PGCs and to later stage female GCs. Since PGCs are the natural precursors to the gametes [24], induction of functional PGC-like cells (PGCLCs) from PSCs is a significant procedure in reconstituting gametes *in vitro*. Mouse epiblast-like cells (EpiLCs) possess cellular characteristics similar to pregastrulating epiblasts and act as appropriate precursors for the induction of mouse PGCLCs [16]. Researchers found 2iLIF medium, which contained LIF and MAPK/GSK3 pathway inhibitors, could enable mouse ESCs to exhibit characteristics similar to the ICM and reveal more efficient germline competency [4, 48]. Mouse ground-state PSCs in 2iLIF medium, with further induction in ActA, *β*FGF, and KSR conditions for 2 days formed mouse EpiLCs. These mouse EpiLCs were further induced under the conditions of BMP4, LIF, SCF, and EGF for 4-6 days to generate mouse PGCLCs [49]. These mouse PGCLCs exhibited analogous transcriptomic and epigenetic profiles comparable to those of E9.5 migratory mouse PGCs *in vivo*. The epigenetic profiles of PGCLCs were evaluated by H3K9me2 and H3K27me3 which represent histone modification and 5mC levels and compared with their expressions during PGCs formation *in vivo*. The results showed that the H3K9me2 and 5mC levels were increased during ESCs differetiating into EpiLCs whereas they decreased significantly during EpiLCs differentiating into PGCLCs. However, the H3K27me3 level was decreased during ESCs differetiating into EpiLCs and was increased during EpiLCs differetiating into PGCLCs. These dynamic regulations are analogous to that of *in vivo* PGC differentiation. Afterward, mouse PGCLCs formed a “reconstituted ovary” through being aggregated with gonadal somatic cells; then, the “reconstituted ovary” was transplanted to the infertile mouse ovarian bursa [4]. The “reconstituted ovary” simulated the female GCs internal milieu *in vivo* and underwent first meiotic division and generated fully grown GV oocytes. These GV oocytes have multiple layers of granulosa and theca cells similar to the fully grown recipient follicles *in vivo*. GV oocytes then underwent *in vitro* maturation (IVM) to be matured into MII oocytes, which could be fertilized through *in vitro* fertilization (IVF) and obtain healthy fertile offspring that bring normal imprinting pattern [4]. Therefore, this was a remarkable achievement in female GCs development from PSCs *in vitro*. However, in this study, reconstituted PGCLCs were transplanted to the infertile mouse ovary bursa, which meant the ensuing oogenesis was not entirely completed *in vitro*. Therefore, researchers tried the first complete *in vitro* reconstitution of mammalian oogenesis from mouse PGCs in a culture system containing estrogen receptor antagonist [50]. Estrogen receptor antagonists improved normal secondary follicles that contain one single primary oocyte inside. *In vitro* oogenesis was completed following three processes including differentiation of primary oocytes through *in vitro* differentiation (IVD), growth of fully grown GV oocytes through *in vitro* growth (IVG), and maturation of MII oocytes through IVM. MII oocytes delivered healthy fertile offspring through IVF [50]. Afterward, mouse PGCLC induction and *in vitro* oogenesis from mouse PGCs referred to in the above studies were combined to reconstitute the whole process of mouse oocyte formation *in vitro*. PSCs were first differentiated to EpiLCs and generated PGCLCs in BMP4, LIF, SCF, and EGF conditions. Then, PGCLCs were aggregated into “reconstituted ovary” with E12.5 gonadal somatic cells and further generated MII oocytes through the IVD, IVG, and IVM process. These MII oocytes were fertilized with wild sperms *in vitro* and delivered healthy fertile offspring that have comparable weights, survival rates, fertility, and gene expression dynamics to wild types (Figure 3) [5]. Besides, the blastocyst from the fertilized PSCs-derived oocytes was evidenced to generate ESCs that could accomplish the whole female GCs generation. Thus, the mouse female germline cycle was established entirely *in vitro* from PSCs.

In the abovementioned milestone studies, “reconstituted ovary” containing gonadal somatic cells played a critical role in promoting differentiation into further stages [4, 5]. However, studies that induce female GCs without gonadal somatic cells are still useful in that they could reveal female GCs developmental mechanisms. Overexpression of germline-related genes also provided a distinct approach for female GCs induction. Transient overexpression of DAZL, which is essential for germ cell development and differentiation, could inhibit pluripotency genes NANOG expression and promote meiotic progression to oocyte-like cell formation [51]. Simultaneous overexpression of PRDM14 alone or of three germline genes BLIMP1, PRDM14, and TFAP2C could induce germline induction [52]. Overexpression of NANOG alone was also found to induce PGCs formation. In this study, NANOG was found to bind to PRDM14 and BLIMP1 enhancers, indicating NANOG functions upstream of both PRDM14 and BLIMP1 [53]. These TF-based inductions of the germline opened up new possibilities to generate female GCs without cytokines and elucidated the transcription networks more elaborately. Some other researchers expanded PGCLCs through cAMP signal stimulation [54]. Expanded PGCLCs maintained the characteristics of sexually uncommitted PGCs, after which sex differentiation was initiated with the presence of gonadal somatic cells *in vivo*. Then, BMP2 and RA synergistically further induced expanded PGCLCs into primary oocyte-like cells that expressed VASA and SCP3 comparable to E15.5 primary oocytes *in vivo* [54]. Thus, BMP and RA were demonstrated to synergically initiate sex determination without gonadal somatic cells [55]. It might be possible to extend the meiosis even further with extra cytokine exposure. These findings have provided a framework for sex differentiation and meiosis initiation.

As described above, mouse female GCs were recapitulated *in vitro* from PSCs using different approaches [51–53]. This demonstrated mouse PSCs act as an effective source for female GCs regeneration. The correct reconstruction of epigenetic reprogramming that occurred during female GCs formation has drawn attention recently. Considering both oocytes and gonadal somatic cells are originated from the fetal ovary, researchers assumed iPSCs derived from gonadal somatic cells may have germline epigenetic memory more analogous to oocytes than other somatic cell-derived iPSCs [56]. Previously, researchers assumed mouse iPSCs from mouse ovarian granulosa cells could spontaneously differentiate into cells expressing oocyte markers in a higher incidence [57]. Recently, researchers achieved granulosa cell-derived iPSCs with a high germline competency through a chemical approach containing crotonic sodium. These iPSCs were induced into PGCLCs following EpiLCs formation; then, PGCLCs formed the “reconstituted ovary” with E12.5 gonadal somatic cells. PGCLCs underwent normal meiosis and formed GV oocytes that could produce healthy fertile offspring after IVM and IVF treatment. Additionally, the “reconstituted ovary” exhibited endocrine functions, including FSH, E2, and AMH secretion. Thus, this study generated oocytes from germline-derived iPSCs [58]. These improvements provided new iPSCs sources and induction methods for stem cell-derived oocytes.

Collectively, through two decades of efforts, researchers have achieved healthy fertile offspring from MII oocytes induced from mouse PSCs. Both genetic manipulations through overexpressing related genes and environment modification strategies using gonadal somatic cells were successful in generating mouse female GCs from PSCs. The environment modification strategy was mostly welcomed since it could simulate *in vivo* environment [57]. Generating PGCLCs from PSCs through EpiLCs and subsequently combining the PGCLCs with gonadal somatic cells have been accepted as the most effective protocol for mouse female GCs induction [5, 41, 59]. More expanded studies about induction details had been investigated using this induction protocol and revealed further understandings about the genesis mechanism of female GCs, which in turn contributed to improving the culture system and induction efficiency [54, 55, 58, 60].

### 3.2. Female GCs Induction from Human PSCs *In Vitro*

Mouse PSCs-based female GCs induction lay the foundation for human female GCs generation *in vitro* (Table 2). After female GCs induction from mouse ESCs in 2003 [40], researchers detected that human ESCs also could be spontaneously differentiated into EBs in suspension culture and generate putative female GCs that express VASA and SCP3 as well as oocyte marker GDF9. This indicated that human ESCs could spontaneously enter the female germline and undergo meiosis [61]. To promote differentiation, researchers added BMP4 in the differentiation culture and found it could increase the induction efficiency and expressions of VASA and SCP3 compared with spontaneous differentiation [62]. RA supplementation also could enhance human ESCs induced into the oocyte and primordial FLCs that possess similar cellular morphology with *in vivo* counterparts [63]. However, the zona pellucida matrix was not detected in these original studies. The addition of gonadal somatic cells was assumed to promote female GCs induction. When human iPSCs and ESCs were induced with gonadal somatic cells at the initial phase, PGCs expressed increased C-KIT, SSEA1, and VASA [12]. Even though these abovementioned researches generated female GCs that express GCs markers, these studies also displayed lower induction efficiency and insufficient characterization of the generated cells [64, 65].

In mice, overexpression of GCs-specific genes without cytokines provided a new approach for mouse female GCs induction [51, 52]. Therefore, researchers also overexpressed GCs-specific genes in human PSCs to enhance induction efficiency [66–69]. Overexpression of DAZ, DAZL, and BOULE promoted meiosis initiation and formed later stage female GCs that express SCP3 [66–68]. Additionally, the STELLA overexpression with RA induction led to VASA upregulation [69]. However, human PSCs induction efficiency is closely correlated with their pluripotency state [70]. Researchers found conventional human PSCs exhibit primed pluripotency [70] and bear properties more similar to mouse postimplantation and epiblast-derived stem cells (EpiSCs) [71, 72], which essentially lack competence for female GCs fate. Naive human PSCs are prone to response for germline specification signals and possess higher induction efficiency compared to primed human PSCs [70]. If human primed PSCs could be transformed into naive PSCs, mouse PGCLCs induction methods could be directly applied to human PSCs. 4i medium (MAPK, GSK3, p38, and JNK inhibitors) facilitate primed state human PSCs transferred into the naive state [73]. Following mouse PGCLCs methods, naive state human PSCs were preinduced with TGF*β*, *β*FGF, and LIF for 2 days, then achieved human PGCLCs under BMP2/4, LIF, SCF, and EGF conditions for 8 days (Figure 3) [7]. Thus, a robust approach for human PGCLCs was established. In another study, primed human iPSCs were cultured under a feeder-free condition with *β*FGF, then stimulated by ActA and a WNT signaling agonist (CHIR99021) for 2 days. The obtained cells expressed pluripotency and mesoderm genes, indicating that they were corresponding to incipient mesoderm-like cells (iMeLCs). Then, iMeLCs were cultured under the GMEM/KSR, BMP4, LIF, SCF, and EGF conditions for 4 days and generated human PGCLCs that correspond to Wk7 human PGCs *in vivo* (Figure 3) [6]. Another team also achieved human PGCLCs differentiation from human PSCs almost at the same time in a concentration-dependent manner. They induced human PSCs into mesodermal-like cells with ActA, *β*FGF, and a low concentration (5 ng/ml) of BMP4, then generated mesodermal-like cells differentiated to human PGCLCs with a high concentration (100 ng/ml) of BMP4 [36]. Thus, the successful derivation of human PGCLCs *in vitro* enabled researchers to reveal more female GCs differentiation mechanism to reestablish them *in vitro*.

Further induction of mouse PGCLCs was continued with the presence of E12.5 gonadal somatic cells; however, human gonadal somatic cells are hard to be acquired from early embryos. Therefore, an alternative approach that does not need the human embryonic gonadal somatic cells was required to enhance *in vitro* differentiation. Overexpression of DAZL and BOULE enabled human ESCs to exit the pluripotent state and enter meiosis. Then, the subsequent addition of GDF9 and BMP15 enhanced the FLCs induction that expresses ZP2 and NOBOX [74]. Thus, they provided a significant new model for generating FLCs from human ESCs without gonadal somatic cells. However, to establish human female GCs *in vitro*, gonadal somatic cells are indispensable considering *in vivo* female GCs development. Considering the restrictions on human embryonic gonadal somatic cell acquisition, in a recent study, researchers substituted human embryonic gonadal somatic cells with that of mice [75]. Human PGCLCs were aggregated with mouse gonadal somatic cells to form a “xenogenic reconstituted ovary.” In the “xenogenic reconstituted ovary,” human PGCLCs were induced for 121 days (Figure 3) [8]. In the generated cells, early PGC genes BLIMP1, TFAP2C, SOX17, and NANOS3 were downregulated; DAZL, VASA, and RA responsive genes STR8 and SCP3 were further upregulated, whereas key meiosis genes DMC1, *γ*H2AX, or SCP1 were not adequately upregulated. Therefore, these generated cells in the “xenogenic reconstituted ovary” were corresponding to RA-responsive female GCs and oogonia, indicating that these cells were in a state corresponding to meiotic entry signals but not yet initiated meiotic recombination. Additionally, these oogonia-like cells expressed similar DNA demethylation and imprint erasure characteristics with oogonia at Wk10 *in vivo*. These results indicated that mouse gonadal somatic cells had provided a suitable environment for human PGCLCs to enter sex differentiation. However, human PGCLCs did not enter meiosis after cultivation up to 121 days, during which human PGCs would have completed meiosis I *in vivo* [1]. This might be because the signals generated from mouse gonadal somatic cells are inadequate to initiate meiosis. Theoretically, human PSC-induced human gonadal somatic cells would be an alternative to human fetal gonadal somatic cells and could further enhance human PGCLCs to postmeiotic phase. In previous studies, human granulosa cells that induce from human iPSCs were transplanted into POF mouse ovaries. They were found to improve ovarian maturation and enhance follicular growth through hormone secretion [76]. Recently, other researchers also derived granulosa cells from human iPSCs through EB formation, and these granulosa cells also contribute to estradiol synthesis *in vitro* [77]. Next, whether these human iPSCs-derived granulosa cells could serve as human gonadal somatic cells and aggregate with PGCLCs to prompt further differentiation and support oocyte formation needs to be investigated.

Remarkably, in recent years, ovarian-related pluripotent stem cells have been discovered in the ovary surface epithelium. Initially, small round cells with diameters from 2 to 4 *μ*m were derived from the ovary surface epithelium of women who had no natural oocytes and follicles. These cells expressed early embryonic markers SSEA4, OCT4, NANOG, SOX2, and C-KIT and possessed a robust proliferation ability. Therefore, they were named as very small embryonic-like stem cells (VSELs) and considered as new stem cell sources for oocytes. These VSELs could be differentiated into oocyte-like cells with diameters of 80–95 *μ*m at day 20, which is comparable to human oocytes that could be used to fertilize. They also expressed VASA and ZP2 and even formed a zona pellucida-like structure. However, meiotic marker SCP3 was not detected in these cells, indicating that they were immature compared with their *in vivo* counterparts [78]. Afterward, another study also established the VSELs in menopausal women ovaries, and these VSELs were evidenced to spontaneously differentiate into oocyte-like cells with zona pellucida-like structures and protrude polar body-like structures. However, the fertilization functionality of these oocyte-like cells had not been tested [79]. Recently, a study showed oocyte-like cells from premature ovarian failure patients' VSELs. These cells exhibited zona pellucida-like structures and could react to sperm. In turn, the sperm could recognize the oocyte-like cells and bound to them strongly. However, these oocyte-like cells did not express ZP1 and ZP2 in spite of the presence of zona pellucida-like structures. Therefore, regardless of the reaction to sperms, these oocyte-like cells could not be a substitute for fully functional oocytes *in vivo* yet [80]. Further precise investigations are still needed to achieve more matured functional oocytes from VSELs.

In summary, similar to mouse PGCLCs, human oogonia-like cells have been successfully achieved through “xenogenic reconstituted ovary” from iPSCs [8]. The multistage systemic protocols for human PGCLCs generation are the remarkable methods in this field over these years [6, 7]. VSELs that contributed to sperm reactive oocyte-like cells have provided a new prospect for functional oocyte formation. Even though fully functional oocytes for clinical researches are still at a distance, these attempts and improvements have provided accessible approaches to study female GCs-specific genes, PGCs migration pathway, sex differentiation, and meiotic initiation. Now, highly efficient and reproducible protocols for PGCLCs differentiation into genetically and epigenetically healthy, patient-specific oocytes are in demand.

## 4. Current Challenges and Future Perspectives

Mouse and human female GCs induction *in vitro* from PSCs achieved significant improvements. It gave us perspectives when they also aroused some challenges in PSCs sources, female GCs development progression, induction culture conditions, and ethical issues.

Firstly, a key issue to be investigated is the stem cell characteristics which are associated with the robustness of induction. ESCs and iPSCs both have the competence for female GCs reconstitutions *in vitro*. Especially, iPSCs are more welcomed because of less harmful access and less immune rejection [10]. Researchers demonstrated that different iPSC lines derived from distinct cell types possess different female GCs fate competency [81]. After the researchers demonstrated mouse oocytes from granulosa cell-derived iPSCs possess a higher germline competency than other cell lines, the certain human granulosa cells discarded after IVF were also considered as a more permissive cell source for iPSCs to generate oocytes [58]. Human iPSCs could provide patient-specific PSCs which could be used to investigate disease-specific pathogenesis *in vitro* [26, 82, 83]. Recently, a study established human iPSCs in 4i medium from patients with premature ovarian insufficiency. Patient-specific iPSCs were preinduced with *β*FGF and TGF for 4 days; then, unlike previous studies [7], the DNA methyltransferase inhibitor was added on day 5. Then, generated cells were further induced into human PGCLCs with BMP2/4, LIF, SCF, EGF, and GMEM/KSR supplement. Compared to the previous human PGCLCs induction methods, the addition of DNA methyltransferase inhibitor enhanced human PGCLCs induction. Thus, they provided a complementary way for human PGC differentiation from patient-specific iPSCs [82]. Furthermore, the PSCs pluripotent state was also considered as an important factor during induction. Previously, human-primed PSCs were maintained in a 4i medium for 2 weeks to obtain naive pluripotency, but studies showed naive human PSCs maintained in the 4i medium for a prolonged time had chromosomal instability and structural anomalies [84]. When researchers cultured human naive PSCs in 4i medium for 3 days instead of 2 weeks, they gained more stable human naive PSCs that could be induced into PGCLCs through EB formation with a high yield in 13 days [85]. Therefore, the efforts on coordinating human PSCs pluripotency state to establish more stable PGCLCs are also an important issue on GCs induction.

Secondly, female GCs development progression has not been clearly revealed yet. The mechanisms underlying female GCs differentiation after Wk3 have been acquired largely; however, the investigations of early embryos before Wk2 remained inadequate for a long time. Recently, a genome-wide DNA methylation map during human preimplantation development was revealed by single-cell chromatin overall omic-scale landscape sequencing in human preimplantation embryos [86]. This gives us a hint about the human PGCs origin before Wk2. Single-cell RNA-seq technology which was recently used to analyze transcriptomic mechanisms among different stage spermatids could be used on PGCs to further analyze PGCs migration, proliferation, and differentiation [87]. Furthermore, researchers used single-cell transcriptome and epigenome sequencing technologies and divided female fetal GCs into three sequential differentiation stages, including the RA responsive stage, the meiotic prophase stage, and the primordial follicle stage. Different stages correspond to distinct gene expressions and epigenetic regulations [26]. These distinct epigenetic regulatory networks of female GCs at sequential developmental phases could be studied through the genome-wide DNA methylation and chromatin accessibility using single-cell resolution [88]. These efforts on female GCs development mechanisms would contribute to a more efficient and stable female GC induction *in vitro*.

Thirdly, the culture condition would also affect the survival of female GCs. Although human PGCLCs had been recapitulated *in vitro*, U-bottom 96 plates or other similar plates used in these studies limited the scale production of human PGCLCs production [6–8]. Recently, a new modified system of methylcellulose-based 3D induction system combined with low-cell attachment plates was reported to produce human PGCLCs from human PSCs at a large scale, with similar gene expression and epigenetic modification profiles to human PGCs [88]. Besides the 3D induction system, 3D bioprosthetic ovaries were also confirmed to provide 3D support for oocyte cultivation. Pore geometry of 3D-printed microporous hydrogel scaffold affected the mouse ovarian follicle survival through the intrafollicular signaling and the ovarian microenvironment [89]. When a 3D-printed scaffold with ovarian follicles was transplanted to a surgically sterilized mouse, they could give birth to healthy fertile offspring. In the next step, whether a 3D-printed ovary could provide an environment more analogous to *in vivo* ovarian microenvironment for PSCs induction needs to be investigated in the future.

Finally, the ethical issues of reproductive medicine have always attracted attention from the scientific community and the public. The establishment of iPSCs has eliminated the concerns about embryo destruction [10], and there are no serious abnormalities in the offspring from mouse PSCs [5]. However, when it comes to the human female GCs induction, concerns about stem cell sources, technology safety, the clinical application of generated cells, and the epigenetic regulation of offspring still exist widely.

Although complete oocytes from human PSCs have not been achieved in a dish yet, it may theoretically possible to integrate the existing methods such as human PGCLCs induction, granulosa cell induction from iPSCs, GV oocyte formation, IVM treatments to form MII oocytes *in vitro*. If it is possible, this would create a great promise for understanding the complex biological process of oocyte development, also would provide a unique cell model for infertility-related drug testing, and even become a more plausible prospect for treating infertility.

## Figures and Tables

**Table 1:** Mouse female GCs differentiation from PSCs *in vitro*.

Cell types	Main induction methods	Achievements		Journal, year (reference)
		Generated cells	Characterization of generated cells	
ESCs	Spontaneous differentiation Suspension culture	FLCs Oocyte-like cells	Morphology Marker expressions (ZP2, ZP3, and FIG*α*) Estradiol secretion Estrogen biosynthesis	Science, 2003 [40]
ESCs	CM from testicular cell Suspension culture	Oocyte-like cells	Morphology Marker expressions (SCP3, ZP3, and FIG*α*)	Stem cells, 2006 [42]
ESCs	(i) Spontaneous differentiation Suspension culture (ii) Coculture with gonadal cells Adherent and suspension culture	PGCs Oocyte like cells	Marker expressions (ZP3, FIG*α*, and GDF9)	Differentiation, 2007 [43]
ESCs	DAZL overexpression Suspension culture	FLCs Oocyte-like cells	Morphology Marker expressions (ZP1, ZP2, ZP3, and GDF9) Parthenogenesis activation	J Mol Cell Biol2009 [51]
ESCs iPSCs	(i) 2i (MAPK and GSK3 inhibitors), LIF, ActA, and bFGF Adherent culture (ii) LIF, SCF, BMP, and EGF Suspension culture	EpiLCs PGCLCs	Morphology Marker expressions (Blimp1 and STELLA) Global transcription profiles Epigenetic analysis	Cell, 2011 [49]
ESCs iPSCs	(i) Coculture with gonadal cells (ii) *In vivo* transplantation into mouse (iii) IVM and IVF	PGCLCs GV oocytes Fertile GCs	Morphology Marker expressions (BLIMP1 and PRDM14) Live offspring delivery	Science, 2012 [4]
ESCs iPSCs	(i) bFGF and ActA Adherent culture (ii) Overexpression of PRDM14 or PRDM1, PRDM14, and TFAP2C Suspension culture	EpiLCs PGCLCs	Morphology Marker expressions (BLIMP1 and STELLA) Global transcription profiles Epigenetic analysis	Nature, 2013 [52]
ESCs iPSCs	(i) bFGF and ActA Adherent culture (ii) NANOG overexpression Suspension culture	EpiLCs PGCLCs	Morphology Marker expressions (BLIMP1 and NANOS3) Global transcription profiles Epigenetic analysis	Nature, 2016 [53]
ESCs iPSCs	(i) Coculture with gonadal cells (ii) IVD, IVG, IVM, and IVF	PGCLCs MII oocytes Fertile GCs	Morphology Marker expressions (DAZL and STELLA) Global transcription profiles Polar body extrusion Live offspring delivery	Nature, 2016 [5]
ESCs iPSCs	(i) 2i (MAPK and GSK3 inhibitors), LIF, ActA, and bFGF Adherent culture (ii) LIF, SCF, BMP, and EGF Suspension culture (ii) BMP2 and RA Adherent culture	PGCLCs Primary oocytes	Morphology Marker expressions (STRA8, SCP3, and NOBOX) Transcriptome dynamics Premeiotic DNA replication	The EMBO Journal, 2017 [55]
iPSCs	(i) iPSCs from granulosa cells (ii) PGCLCs coculture with gonadal cells (iii) IVD, IVG, IVM, and IVF	PGCLCs MII oocytes Fertile GCs	Morphology Marker expressions (BLIMP1, DAZL, and VASA) Telomere elongation Endocrine activity of FSH, E2, and AMH Live offspring delivery	Cell Rep, 2019 [58]

ESCs: embryonic stem cells; iPSCs: induced pluripotent stem cells; GCs: germ cells; EpiLCs: epiblast-like cells; PGCs: primordial germ cells; PGCLCs: primordial germ cell-like cells; FLCs: follicle-like cells; GV oocytes: germinal vesicle oocytes; MII oocytes: meiosis II oocytes; CM: conditioned medium; IVD: *in vitro* differentiation; IVG: *in vitro* growth; IVM: *in vitro* maturation; IVF: *in vitro* fertilization.

**Table 2:** Human female GCs differentiation from PSCs *in vitro.*

Cell types	Main induction methods	Achievements		Journal, year (reference)
		Generated cells	Characterization of generated cells	
ESCs	Spontaneous differentiation Suspension culture	Oocyte-like cells	Morphology Marker expressions (SCP1, SCP3, and GDF9)	Hum Mol Genet, 2004 [61]
ESCs	BMP4 Suspension culture	PGCs	Morphology Marker expressions (VASA and SCP3)	Stem cells dev, 2006 [62]
VSELs	Spontaneous differentiation Suspension culture	Oocyte-like cells	Morphology Marker expressions (C-KIT, VASA, and ZP2)	Differentiation, 2008 [78]
ESCs iPSCs	Coculture with fetal gonadal cells Adherent culture	PGCs	Morphology Marker expressions (DAZL, VASA, and SSEA1)	Stem Cells, 2009 [12]
ESCs	VASA overexpression BMP4, BMP7, and BMP8b Adherent culture	PGCs	Morphology Marker expressions (DAZL, VASA, and SCP3) Epigenetic analysis	Nature, 2009 [66]
ESCs	RA Suspension culture	Oocyte-like cells	Morphology Marker expressions (SSEA1, DAZL, and VASA)	Hum Repro, 2009 [63]
iPSCs	Overexpression of DAZL and BOULE BMP4, BMP7, and BMP8b Adherent culture	PGCs	Morphology Marker expressions (STELLA and DMC1) Elongated SC formation	Human Mol Genet, 2011 [68]
VSELs	Spontaneous differentiation Adherent culture	Oocyte-like cells	Morphology Marker expressions (DAZL, ZP4, and GDF9)	Stem Cells Dev, 2011 [79]
ESCs iPSCs	VASA overexpression Adherent culture	PGCs Postmeiotic GCs	Morphology Marker expressions (GCNF, LHR, and ZP2) SCP formation analysis Epigenetic analysis	Stem Cells, 2012 [67]
ESCs	STELLA overexpression RA Adherent culture	PGCs	Marker expressions (VASA, SCP3, and SOX17)	PloS one, 2013 [69]
ESCs iPSCs	(i) 4i (MAPK, GSK3, P38, and JNK inhibitors), LIF, TGF*β,* and bFGF Adherent culture (ii) BMP2/4, LIF, SCF, and EGF Suspension culture	PGCLCs	Morphology Marker expressions (BLIMP1 and STELLA) Global transcription profiles Epigenetic analysis	Cell, 2015 [7]
iPSCs	(i) ActA and GSK3b inhibitor Adherent culture (ii) GMEM/KSR, BMP4, LIF, SCF, and EGF Suspension culture	iMeLCs PGCLCs	Morphology Marker expressions (PRDM14 andSOX17) Global transcription profiles Epigenetic analysis	Cell stem cell, 2015 [6]
ESCs iPSCs	(i) ActA, bFGF, and BMP4 (5 ng/ml) Adherent culture (ii) Lif and BMP4 (100 ng/ml) Suspension culture	Mesodermal-like cells PGCLCs	Morphology Marker expressions (BLIMP1 and STELLA) global transcription profiles Epigenetic analysis	EMBO J, 2015 [36]
ESCs	Overexpression of DAZL and BOULE GDF9 and BMP15 Adherent culture	FLCs	Morphology Marker expressions (ZP2, NOBOX, and AMH) Global transcription profiles Estradiol secretion	Nat commun, 2017 [74]
VSELs	Follicular fluid “serum” medium Adherent culture	Oocyte-like cells	Morphology Marker expression (ZP1-3)	Stem Cell Rev Rep, 2018 [80]
iPSCs	Coculture with mouse gonadal cells Suspension culture	PGCLCs Oogonia-like cells	Morphology Marker expressions (SCP3, REC8 and STRA8) Transcriptome dynamics Epigenetic analysis X chromosome activity	Science, 2018 [8]

ESCs: embryonic stem cells; iPSCs: induced pluripotent stem cells; VSELs: very small embryonic-like stem cells; iMeLCs: incipient mesoderm-like cells; PGCs: primordial germ cells; PGCLCs: primordial germ cell-like cells; FLCs: follicle-like cells; SCP: synaptonemal complex protein.
